# Thyroid hormone therapy initiation after hemithyroidectomy: treatment burden, timing, and predictors in a population-based cohort

**DOI:** 10.1007/s12020-026-04707-3

**Published:** 2026-07-10

**Authors:** Shmuel Wechsler, Tal Marom, Bernice Oberman, Avital Fellner, Yael Reichenberg, Jacob Pitaro, Limor Muallem Kalmovich

**Affiliations:** 1 Department of Otolaryngology–Head and Neck Surgery, Shamir Medical Center, Beer Yaakov, Israel; 2 Gray Faculty of Medical and Health Sciences, Tel Aviv University, Tel Aviv, Israel; 3 Clalit Health Services, Dan Petah-Tikvah District, Ramat Gan, Israel; 4 Department of Otolaryngology–Head and Neck Surgery, Samson Ashdod University Hospital, Ashdod, Israel; 5 Faculty of Health Sciences, Ben-Gurion University of the Negev, Beer Sheva, Israel

**Keywords:** Hemithyroidectomy, Thyroid lobectomy, Levothyroxine, Thyroid hormone therapy, Hypothyroidism, Treatment initiation

## Abstract

**Purpose:**

To determine the two-year burden, timing, and predictors of thyroid hormone therapy initiation after hemithyroidectomy in previously euthyroid adults.

**Methods:**

Retrospective population-based cohort study using de-identified electronic health record data from Clalit Health Services (2003–2020), extracted through the MDClone research platform. Adults undergoing hemithyroidectomy with preoperative TSH < 5.0 mIU/L, no preoperative thyroid hormone therapy, and at least two years of follow-up were included. The primary endpoint was first levothyroxine dispensing or overt biochemical hypothyroidism within 24 months.

**Results:**

Among 8,467 eligible patients, 3,362 (39.7%) reached the endpoint within 24 months: 2,179 (25.7%) by 4 months and 3,100 (36.6%) by 12 months. Extended follow-up identified 558 additional initiations (cumulative 46.3%). Treatment initiation was markedly higher among patients with thyroid cancer (72.7%) than those without (33.4%). The strongest multivariable predictors were preoperative TSH (OR 1.55 per 1 mIU/L; 95% CI, 1.47–1.64) and thyroid cancer (OR 4.99; 95% CI, 4.29–5.81).

**Conclusion:**

Thyroid hormone therapy initiation is common after hemithyroidectomy, affecting nearly 40% of previously euthyroid adults within two years. Preoperative TSH and thyroid cancer identify high-burden subgroups and should inform preoperative counseling when hemithyroidectomy is chosen to preserve endogenous thyroid function.

## Introduction

Hemithyroidectomy is increasingly used for benign thyroid disease and selected low-risk differentiated thyroid cancers because it preserves the contralateral thyroid lobe and reduces the risks associated with total thyroidectomy, including hypoparathyroidism and bilateral recurrent laryngeal nerve injury [1–4]. A central clinical rationale for choosing hemithyroidectomy is the possibility of maintaining endogenous thyroid function and avoiding postoperative thyroid hormone therapy.

Post-hemithyroidectomy thyroid dysfunction has been extensively studied. Systematic reviews and meta-analyses report that approximately 20–40% of patients develop laboratory-defined hypothyroidism, with preoperative TSH and thyroid autoimmunity consistently emerging as key predictors [5–7]. Large database and registry studies have extended these observations across different health systems: Hu et al. evaluated postoperative thyroid hormone supplementation after thyroid lobectomy in a national United States claims cohort [8], and Villar-del-Moral et al. documented early thyroxine replacement and practice variation across European Eurocrine centers [9].

These studies establish that biochemical hypothyroidism is common after hemithyroidectomy. A related but distinct clinical question is whether the patient will actually start thyroid hormone therapy after surgery, the practical outcome that hemithyroidectomy is partly chosen to avoid. This distinction is clinically relevant because postoperative thyroid dysfunction after lobectomy has been associated with patient-reported quality-of-life outcomes [10]. Treatment initiation integrates biochemical thyroid dysfunction with real-world clinical decision-making, including symptoms, cancer-related TSH management, clinician thresholds, and follow-up intensity. We therefore evaluated thyroid hormone therapy initiation as a pragmatic postoperative endocrine endpoint in a large population-based cohort of previously euthyroid adults.

## Materials and methods

### Study design and data source

This retrospective cohort study used data from the Clalit Health Services (CHS) electronic health records, extracted through the MDClone research platform. CHS is Israel’s largest integrated healthcare organization, insuring more than 4.8 million individuals, with linked hospital, community, laboratory, and pharmacy records [11]. Data were extracted as de-identified real-world patient-level records; patient identifiers were removed and dates were de-identified according to institutional privacy procedures. The present study did not use MDClone’s synthetic-data generation model. The study was approved by the CHS Institutional Review Board (0052-23-COM), and informed consent was waived.

## Study population

Adults aged 18 years or older who underwent hemithyroidectomy between January 2003 and December 2020 were identified using ICD-9 procedure codes. Inclusion required CHS membership and at least two years of postoperative follow-up. Patients were excluded if they had prior thyroid or neck surgery, preoperative thyroid hormone therapy, Lugol treatment, or preoperative TSH > 5.0 mIU/L within six months before surgery.

## Variables

Extracted variables included age, sex, ethnicity, socioeconomic status, area of residence, comorbidities, preoperative thyroid diagnoses, and preoperative laboratory values. Comorbidities included hypertension, diabetes mellitus, chronic obstructive pulmonary disease, and ischemic heart disease. Preoperative thyroid diagnoses included thyroid cancer, Hashimoto thyroiditis, Graves’ disease, and goiter/nodular thyroid disease. Socioeconomic status and residential peripherality were derived from Israeli Central Bureau of Statistics indices [12]. Preoperative laboratory values were defined as the most recent test within six months before surgery.

## Endpoint definition

The primary endpoint was thyroid hormone therapy initiation within 24 months, defined as first documented dispensing of levothyroxine or combination thyroid hormone therapy (ATC codes H03AA01–H03AA05), or overt biochemical hypothyroidism (TSH > 5.0 mIU/L with free T4 < 10 pmol/L at least one month after surgery). An exploratory extended follow-up analysis estimated cumulative treatment initiation after 24 months. Treatment initiation was not interpreted as permanent therapy, treatment persistence, adherence, or lifelong thyroid hormone dependence.

### Statistical analysis

Categorical variables were compared using chi-square tests and continuous variables using Mann–Whitney U tests. Multivariable logistic regression was performed using stepwise selection based on the Akaike Information Criterion. A model including preoperative TSH as a continuous covariate was fitted in the subset with available preoperative TSH data (*n* = 6,239). A calendar-period analysis compared 24-month treatment-initiation rates across three surgical eras (2003–2009, 2010–2015, 2016–2020). Results are reported as odds ratios with 95% confidence intervals. Analyses were performed in R version 4.2.3.

## Results

### Cohort characteristics

**Fig. 1:**
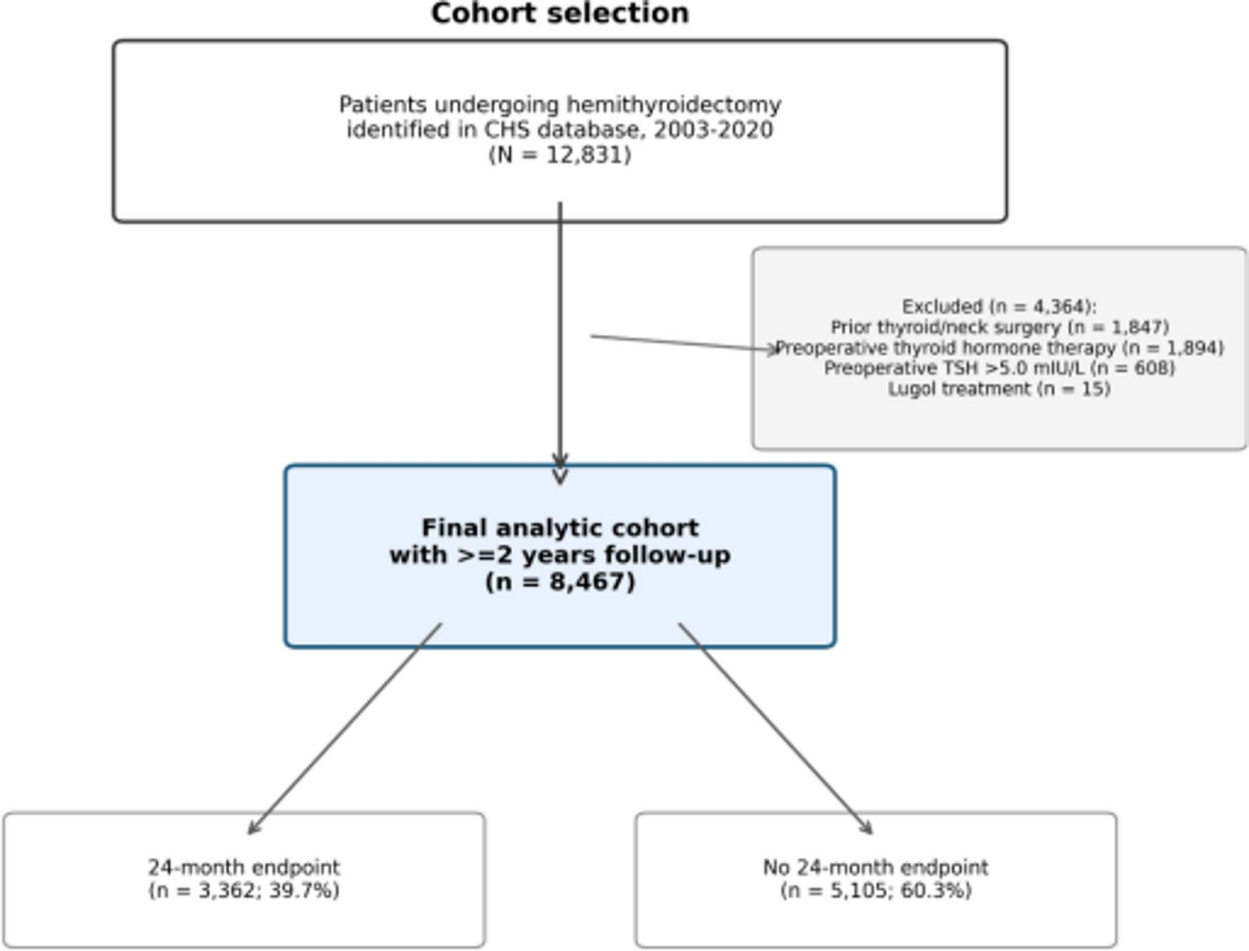
Cohort selection flow diagram. Patients undergoing hemithyroidectomy were identified from the Clalit Health Services database between 2003 and 2020. The final analytic cohort included 8,467 adults, of whom 3,362 reached the 24-month endpoint and 5,105 did not

Among 12,831 patients identified as having undergone hemithyroidectomy with at least two years of follow-up, 8,467 met inclusion criteria after exclusion of patients with prior thyroid or neck surgery (*n* = 1,847), preoperative TSH > 5.0 mIU/L (*n* = 608), preoperative thyroid hormone therapy (*n* = 1,894), or Lugol treatment (*n* = 15) (Fig. 1). The cohort was predominantly female (78.1%). Median preoperative TSH was 1.46 mIU/L (IQR, 0.90–2.21). Thyroid cancer was present in 1,352 patients (16.0%). Baseline characteristics by 24-month endpoint status are shown in Table 1.

**Table 1:** Baseline characteristics by 24-month endpoint status

Characteristic	Overall (*N* = 8,467)	No endpoint (*n* = 5,105)	Endpoint (*n* = 3,362)	*p*
Female sex	6,609 (78.1)	3,942 (77.2)	2,667 (79.3)	0.02
Male sex	1,858 (21.9)	1,163 (22.8)	695 (20.7)	
Age < 40 years	1,816 (21.4)	1,003 (19.6)	813 (24.2)	< 0.001
Age 40–59 years	3,327 (39.3)	1,919 (37.6)	1,408 (41.9)	
Age ≥ 60 years	3,324 (39.3)	2,183 (42.8)	1,141 (33.9)	
Hypertension	4,864 (57.4)	3,025 (59.3)	1,839 (54.7)	< 0.001
Diabetes mellitus	2,073 (24.5)	1,354 (26.5)	719 (21.4)	< 0.001
COPD	923 (10.9)	605 (11.9)	318 (9.5)	< 0.001
Ischemic heart disease	1,148 (13.6)	778 (15.2)	370 (11.0)	< 0.001
Residence – center	3,801 (44.9)	2,399 (47.0)	1,402 (41.7)	< 0.001
Residence – middle	2,890 (34.1)	1,694 (33.2)	1,196 (35.6)	
Residence – periphery	1,750 (20.7)	998 (19.5)	752 (22.4)	
Thyroid cancer	1,352 (16.0)	369 (7.2)	983 (29.2)	< 0.001
No thyroid cancer	7,115 (84.0)	4,736 (92.8)	2,379 (70.8)	
Hashimoto thyroiditis	116 (1.4)	59 (1.2)	57 (1.7)	0.04
Graves disease	39 (0.5)	14 (0.3)	25 (0.7)	0.003
Preop TSH, median (IQR)	1.46 (0.90–2.21)	1.29 (0.79–1.92)	1.75 (1.08–2.55)	< 0.001

Values are n (%) unless otherwise indicated. Endpoint: thyroid hormone therapy initiation or overt biochemical hypothyroidism within 24 months. COPD, chronic obstructive pulmonary disease; IQR, interquartile range; TSH, thyroid-stimulating hormone.

### Treatment-initiation burden and timing

**Fig. 2:**
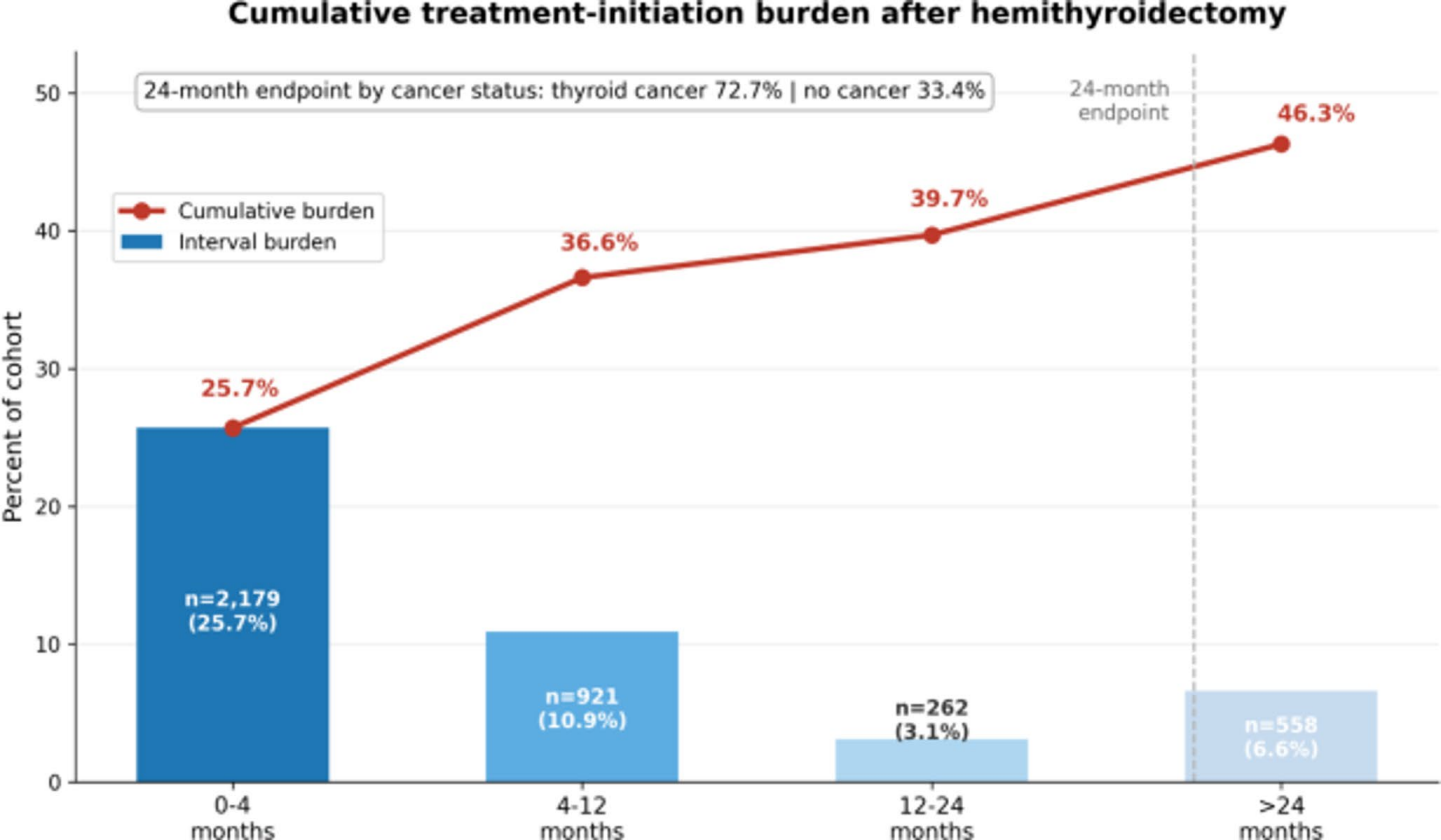
Timing and cumulative burden of the treatment-initiation endpoint after hemithyroidectomy. Bars show the proportion of the cohort reaching the endpoint during each postoperative interval, and the line shows cumulative burden. The 24-month endpoint was reached by 3,362 patients (39.7%); 558 additional patients initiated therapy after 24 months (cumulative 46.3%). The 24-month endpoint was more frequent among patients with thyroid cancer (72.7%) than those without (33.4%)

Within 24 months, 3,362 patients (39.7%) reached the primary endpoint. Most events occurred during the first postoperative year: 2,179 patients (25.7%) reached the endpoint within 0–4 months and 921 (10.9%) between 4 and 12 months. Only 262 patients (3.1%) first reached the endpoint between 12 and 24 months. Over extended follow-up, 558 additional patients initiated therapy after 24 months, increasing the cumulative burden to 3,920 (46.3%) (Fig. 2). Among 24-month endpoints, 3,330 (99.1%) were captured through medication dispensing and 32 (0.9%) were biochemical-only events.

### Treatment initiation by thyroid cancer status

**Fig. 3:**
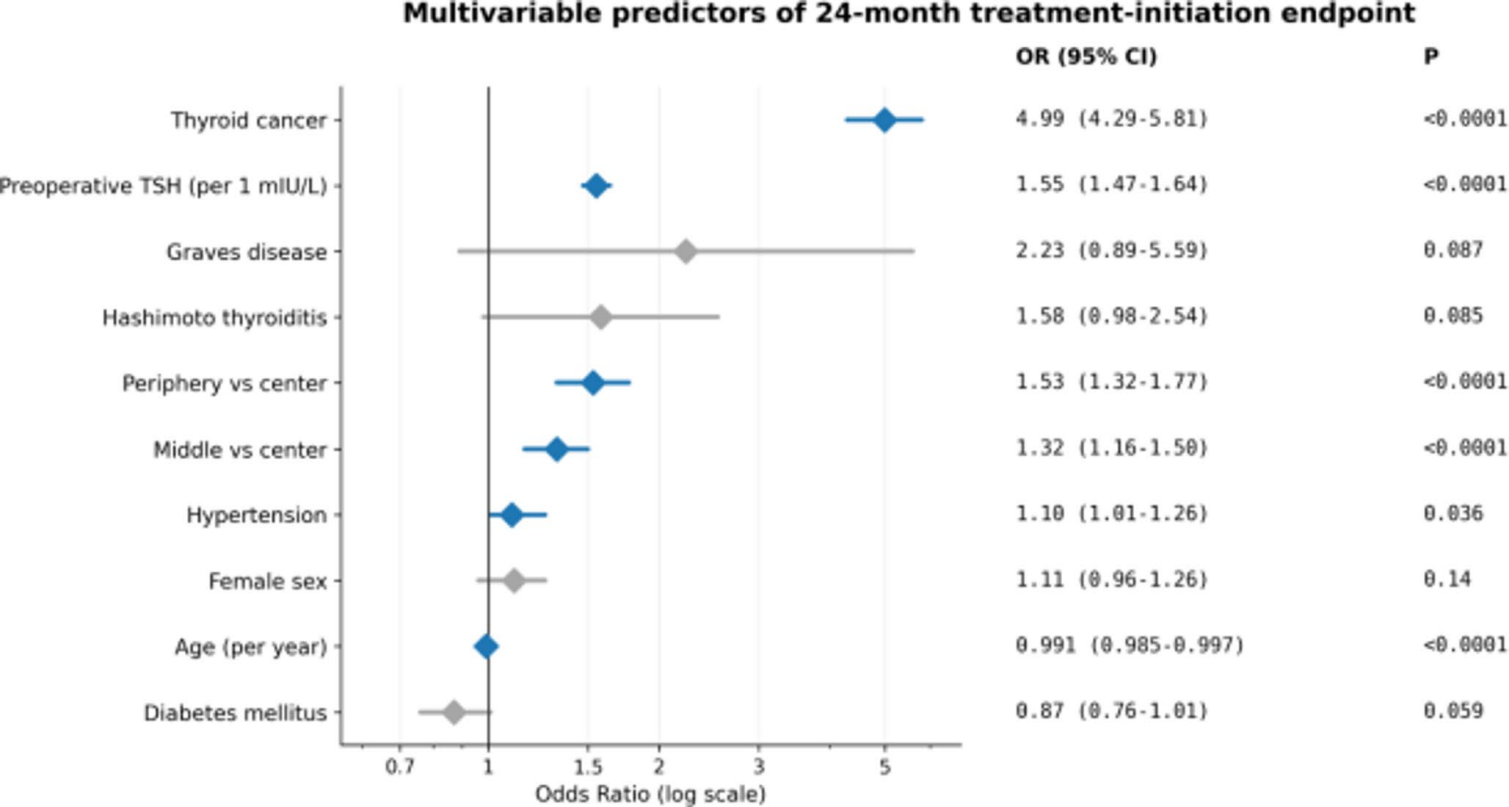
Multivariable predictors of the 24-month treatment-initiation endpoint. Odds ratios and 95% confidence intervals from the logistic regression model including preoperative TSH (*n* = 6,239). Blue markers indicate statistically significant predictors; gray markers indicate non-significant variables. The vertical line denotes OR = 1.0

Thyroid cancer was associated with a markedly higher 24-month treatment-initiation burden: 983 of 1,352 patients with thyroid cancer (72.7%) reached the endpoint compared with 2,379 of 7,115 patients without thyroid cancer (33.4%; *p* < 0.001). This association persisted in the multivariable model (OR, 4.99; 95% CI, 4.29–5.81) (Fig. 3).

### Predictors of 24-month treatment initiation

In the multivariable model including preoperative TSH, the strongest predictors were higher preoperative TSH (OR, 1.55 per 1 mIU/L; 95% CI, 1.47–1.64; *p* < 0.0001) and thyroid cancer (OR, 4.99; 95% CI, 4.29–5.81; *p* < 0.0001). Each 2 mIU/L higher preoperative TSH corresponded to approximately 2.4-fold higher odds. Younger age, residence in middle or peripheral regions, and hypertension were also independently associated with treatment initiation (Fig. 3).

### Calendar-period analysis

The 24-month treatment-initiation rate was 37.2% (1,344/3,610) for patients operated during 2003–2009, 42.1% (1,010/2,397) for 2010–2015, and 41.0% (1,008/2,460) for 2016–2020. No clear reduction in treatment initiation was observed following the 2015 ATA guideline update. The proportion of patients initiating therapy after 24 months was lower in the most recent period (3.0% vs. 9.3% in 2003–2009), likely reflecting shorter available extended follow-up rather than a true practice change.

### Descriptive postoperative TSH profiles

Patients without treatment initiation maintained stable median TSH values around 2.2–2.4 mIU/L during the first two postoperative years. Patients who initiated therapy generally had higher TSH values around the corresponding postoperative interval (Table 2). Patients initiating therapy after 24 months had high-normal median TSH values during the first two years (median 3.2–3.5 mIU/L). Missingness of TSH data varied across intervals and groups, ranging from 4% among patients measured around their treatment interval to 67–68% for early postoperative windows in groups initiating therapy later. These profiles were therefore interpreted descriptively and were not used to infer causal treatment indications.

**Table 2:** Descriptive postoperative TSH values (mIU/L) by treatment-initiation timing group

Group	TSH window	Median (p25–p75)	Available *n*	% Missing
No initiation	0–1 mo	2.2 (1.4–3.2)	1,500	67
	1–4 mo	2.4 (1.7–3.4)	2,097	54
	4–12 mo	2.3 (1.5–3.2)	2,773	39
	12–24 mo	2.2 (1.5–3.2)	3,068	33
Initiation 0–4 mo	0–1 mo	4.4 (2.5–8.3)	1,305	40
	1–4 mo	5.7 (2.5–16.6)	1,937	11
	4–12 mo	2.8 (1.2–6.5)	1,971	10
	12–24 mo	1.9 (0.6–3.7)	1,842	15
Initiation 4–12 mo	0–1 mo	3.1 (2.0–4.7)	354	62
	1–4 mo	4.5 (2.9–6.4)	652	29
	4–12 mo	4.8 (2.6–8.3)	882	4
	12–24 mo	2.6 (1.1–4.5)	813	12
Initiation 12–24 mo	0–1 mo	3.3 (2.1–4.7)	85	68
	1–4 mo	3.8 (2.9–5.0)	142	46
	4–12 mo	3.9 (2.5–5.4)	182	31
	12–24 mo	5.3 (3.1–7.7)	252	4
Initiation > 24 mo	0–1 mo	2.8 (1.6–4.3)	183	67
	1–4 mo	3.2 (2.3–4.5)	277	50
	4–12 mo	3.4 (2.2–4.6)	364	35
	12–24 mo	3.5 (2.4–4.8)	420	25

TSH values are median (25th–75th percentile) in mIU/L for available observations at each postoperative interval. Missingness varied across intervals and treatment-initiation groups (range 4–68%). Values in intervals temporally adjacent to treatment initiation had lower missingness. These profiles are descriptive and should not be used to infer the indication for treatment initiation. TSH values obtained after levothyroxine initiation reflect treated levels.

## Discussion

In this population-based cohort of 8,467 previously euthyroid adults, thyroid hormone therapy initiation after hemithyroidectomy was common, early, and clinically substantial. Nearly 40% of patients initiated therapy or met overt biochemical criteria within two years, and the cumulative burden increased to 46% over extended follow-up. The strongest predictors were preoperative TSH and thyroid cancer. These findings quantify a pragmatic endocrine outcome after an operation often selected to preserve thyroid function.

### Treatment initiation as a pragmatic endpoint

Prior work has established that laboratory-defined hypothyroidism after hemithyroidectomy is common [5–7]. Tsur et al. recently reported biochemical hypothyroidism in 52.6% of patients, predominantly subclinical, with a median time to diagnosis of 11.4 months [13]. Our study addresses a complementary endpoint: postoperative thyroid hormone therapy initiation. This endpoint captures a broader clinical process than laboratory diagnosis alone, including biochemical abnormalities, symptoms, cancer-related TSH targets, clinician prescribing thresholds, and follow-up patterns. It is the outcome most directly relevant to preoperative counseling: whether the patient enters thyroid hormone treatment after hemithyroidectomy.

### Thyroid cancer and the treatment-burden paradox

The most prominent subgroup finding was the 72.7% treatment-initiation rate among patients with thyroid cancer, compared with 33.4% among patients without cancer. This difference should not be interpreted solely as biological failure of the remaining thyroid lobe, nor solely as oncologic TSH suppression. In patients with thyroid cancer treated by hemithyroidectomy, clinicians may prescribe levothyroxine for a combination of reasons: true biochemical hypothyroidism, risk-adapted TSH management to maintain low-normal values, or clinician preference in the postoperative oncologic setting [2, 14]. Long-term suppressive therapy to subnormal TSH levels is not routinely required after hemithyroidectomy for low-risk thyroid cancer; therefore, the high treatment-initiation rate in this subgroup should not be equated with TSH suppression alone. The postoperative TSH profiles support this interpretation, as patients who initiated therapy generally had higher TSH values around the relevant interval, suggesting that genuine thyroid dysfunction contributed to a substantial proportion of treatment decisions. Regardless of mechanism, the practical implication is clear: hemithyroidectomy substantially reduces surgical morbidity compared with total thyroidectomy, but it does not eliminate the likelihood of postoperative thyroid hormone therapy in patients with thyroid cancer.

### Preoperative TSH and risk stratification

Preoperative TSH showed a graded association with treatment initiation, consistent with prior meta-analyses and population-based studies [5–7, 13]. Even within the normal range, higher TSH reflects lower functional reserve and should be incorporated into preoperative counseling. In our model, a patient with preoperative TSH of 3.0 mIU/L had approximately 2.4-fold higher odds of treatment initiation than a patient with TSH of 1.0 mIU/L.

### Geographic variation within a universal healthcare system

Residence outside central regions was independently associated with treatment initiation despite care occurring within a universal integrated healthcare system. Although Israel has universal health coverage, geographic disparities in access to healthcare have been documented, particularly for peripheral regions [15, 16]. The observed geographic variation in treatment initiation may therefore reflect differences in follow-up patterns, specialist access, local prescribing thresholds, or unmeasured socioeconomic factors; however, provider-level data and endocrinology visit frequency were not available in the dataset. Villar-del-Moral et al. demonstrated substantial between-country practice variation in early thyroxine replacement after hemithyroidectomy across Eurocrine centers [9]. Our data suggest that treatment variation may also exist within a single health system.

### Temporal trends and guideline evolution

The study covers a period during which clinical guidelines changed substantially, including the 2015 ATA recommendations expanding indications for thyroid lobectomy and promoting risk-adapted TSH management [2]. In our calendar-period analysis, the 24-month treatment-initiation rate was 37.2% in 2003–2009, 42.1% in 2010–2015, and 41.0% from 2016 onward. We did not observe a clear post-2015 reduction in treatment initiation, consistent with evidence that implementation of ATA guideline changes in routine clinical practice has been neither immediate nor uniform, as previously documented in a survey of endocrinologists and surgeons [17].

### Timing and monitoring implications

The timing data support concentrated surveillance during the first postoperative year: 92% of 24-month endpoints occurred within 12 months. However, the additional 6.6% of patients initiating therapy after 24 months indicates that late treatment initiation also occurs. A practical approach would include routine thyroid function testing during the first year after hemithyroidectomy and continued monitoring for patients with higher preoperative TSH, thyroid cancer, or high-normal postoperative TSH values.

### Limitations

Several limitations should be considered. First, the endpoint captures thyroid hormone therapy initiation, not treatment persistence, dose, adherence, discontinuation, or intended duration; it should not be equated with permanent thyroid hormone dependence. Second, the specific clinical indication for each prescription was unavailable; we could not distinguish replacement therapy for hypothyroidism from levothyroxine prescribed for TSH management in patients with thyroid cancer. Third, the dataset lacked remnant lobe volume, operative laterality, detailed histopathology, and tumor risk category. Although the independent contribution of remnant lobe volume to postoperative thyroid function remains uncertain, these missing variables may have contributed to residual confounding. Fourth, the low coded prevalence of Hashimoto thyroiditis (1.4%) likely reflects underascertainment of autoimmune thyroid disease in administrative data, which may have attenuated its apparent association with treatment initiation in the multivariable model. Fifth, postoperative TSH data had substantial missingness, particularly in intervals not temporally adjacent to treatment initiation, and these profiles were interpreted descriptively. Finally, the observational design cannot distinguish whether geographic differences reflect true biological variation or differences in clinical practice.

In conclusion, thyroid hormone therapy initiation after hemithyroidectomy is common in real-world practice, affecting 39.7% of previously euthyroid adults within two years and 46.3% over extended follow-up. Preoperative TSH and thyroid cancer identify high treatment-burden subgroups. These data should inform preoperative counseling and postoperative monitoring when hemithyroidectomy is chosen to preserve endogenous thyroid function.

## Data Availability

The data supporting the findings of this study were extracted as de-identified real-world patient-level records from the Clalit Health Services electronic health record system through the MDClone research platform. The underlying source data are not publicly available because of institutional data-protection regulations and privacy restrictions. Aggregated data may be made available from the corresponding author upon reasonable request, subject to approval by Clalit Health Services and applicable institutional policies.

## Declarations

### Use of artificial intelligence tools

Generative AI tools were used only for language editing and manuscript formatting. The authors reviewed and approved all text and remain fully responsible for the scientific content.
